# Nest Predation by Commensal Rodents in Urban Bushland Remnants

**DOI:** 10.1371/journal.pone.0156180

**Published:** 2016-06-13

**Authors:** Helen M. Smith, Chris R. Dickman, Peter B. Banks

**Affiliations:** School of Life and Environmental Sciences, The University of Sydney, NSW, Australia; University of Sydney, AUSTRALIA

## Abstract

Exotic predators are a major threat to native wildlife in many parts of the world. Developing and implementing effective strategies to mitigate their effects requires robust quantitative data so that management can be evidence-based, yet in many ecosystems this is missing. Birds in particular have been severely impacted by exotic mammalian predators, and a plethora of studies on islands record predation of bird eggs, fledglings and adults by exotic species such as rodents, stoats and cats. By comparison, few studies have examined nest predation around mainland urban centres which often act as dispersal hubs, especially for commensal species such as rodents. Here, we experimentally examine nest predation rates in habitat patches with varying black rat (*Rattus rattus*) densities in Sydney, Australia and test whether these exotic rats have the effects expected of exotic predators using effect size benchmarks. In the case where black rats have replaced native *Rattus* spp., we expected that black rats, being more arboreal than native *Rattus* spp., would be a significant source of predation on birds because they can readily access the arboreal niche where many birds nest. We tested this idea using above-ground artificial nests to represent those of typical small bird species such as the New Holland honeyeater (*Phylidonyris novaehollandiae*). We found that fewer eggs were depredated by rodents on sites where we removed black rats compared to unmanipulated sites, and that the effect size calculated from the total number of eggs surviving beyond the typical incubation period was similar to that expected for an exotic predator. Our results suggest that, although Australian birds have co-evolved with native *Rattus* species, in the case where black rats have replaced native *Rattus* species, exotic black rats appear to pose an additive source of predation on birds in remnant habitats, most likely due to their ability to climb more efficiently than their native counterparts. Management of these commensal rodents may be necessary to retain urban birdlife.

## Introduction

Exotic species have devastating impacts on wildlife around the globe [1–3], with island avifauna having suffered particularly severe declines and extinctions upon the arrival of exotic predators (e.g. [4, 5]).Nest predation is a major source of reproductive failure in birds [6–9] and provides strong selective pressure that appears to have considerably shaped bird behaviour and life histories (e.g. [10]). Exotic predators often represent functionally novel predator-types, and the predation pressure they exert can lead to the decline or even extinction of susceptible bird species [11]. Exotic mammalian predators such as mustelids, rodents and cats in particular are primary drivers of bird extinctions on islands [4], whereas in more urban regions, other factors such as habitat destruction or fragmentation, and changes to habitat complexity, can cause declines in bird species, as well as exacerbate the threat of nest predation from native and exotic predators [12].

Nest predation by exotic rodents is considered the leading cause of bird species declines worldwide [13] and is a key secondary cause of decline for many other species (see [14]). Indeed, the impacts of exotic rodents on seabird populations via predation of eggs, chicks and fledglings [5] have been confirmed using stable isotope dietary analyses [15] and video footage [16], and have sparked numerous attempts to eradicate the invaders [17, 18]. Unlike natural areas, the processes affecting bird populations in urban areas are often complicated by the redistribution of resources engendered by human activity, as well as by habitat loss and modification; with these pressures even natural levels of predation can become serious [19, 20]. Urban areas provide additional resources that some wildlife species can use, while others decline dramatically. Commensal rodents are among the species that effectively exploit urban resources [21] and, since island birds often suffer severe predation on nests and fledglings from exotic rodents, we could expect that birds inhabiting remnant urban habitats also are susceptible to predation from commensal rodents.

Urban regions support many opportunistic and generalist predators [22–24], and only some types of birds can co-exist with these predators in dramatically modified urban habitat remnants [24, 25]. Highly modified urban hotspots often provide additional food resources for some native and exotic species, and this type of resource subsidy can lead to increases in predator abundance [26–28], with ‘spillover’ occurring into adjacent natural areas [21]. This may place additional predation pressure from native and/or exotic predators on prey species in urban remnants, presumably with consequences for prey population survival. While the addition of human resources can decouple predator-prey relationships in urban areas, in bushland remnants or bushland surrounding the urban zone, we could expect increased predation pressure and marked declines in nest survival if predators are encroaching into urban remnants and/or surrounding bushland.

In this paper we examine the impacts of exotic black rats (*Rattus rattus*) on bird nest survival in peri-urban bushland remnants of Sydney, Australia’s largest and oldest city. We measure rodent nest predation in a system where black rats have replaced their native bush rat (*Rattus fuscipes*) counterparts. Our system is not measuring the additional effect of black rats on top of the natural (historical) system, but rather the impacts of the exotic black rat when it replaces a native one. Native *Rattus* species first arrived in Australia 1–2 million years ago [29], and Australian birds have co-existed with these rodents since this time. If these native rodents prey upon native birds, as at least some species appear to do [30, 31], their long-standing co-existence should have selected for effective anti-predator defence strategies in birds. By comparison, commensal *Rattus* species such as the black rat have only been present in Australia for 200+ years, having arrived as stowaways with the first fleet in 1788 and established in human settlements thereafter [32]. Exotic black rats are physically similar to their native Australian counterpart, the bush rat, although black rats appear to fare better in human-modified landscapes whereas bush rats hold their ground in natural habitat [33, 34]. In eastern Australia bush rats and black rats are competitively symmetrical (i.e. both species are able to defend territory and resources from the other) where they co-occur [35, 36], but in the Sydney region historical events and habitat fragmentation have led to local extinctions of bush rats and other native small mammals. Now black rats dominate most of the urban remnants of Sydney's foreshore [37]. Our study system is different from that represented by a predator-free island: bird species have co-evolved with egg predators; however, introduced black rats are functionally novel predators in the study system owing to their partly arboreal habits compared to the native rodents they have replaced. Thus black rats pose different, new and potentially additive threats to native bird species.

Rodent abundance and/or activity is often associated positively with nest predation rates [38, 39], and experiments using artificial nests in Australia have shown that black rats attack both arboreal [40, 41] and ground-nesting birds [42]. Rat droppings on the rim of real nests suggest that eggs, nestlings or fledglings may be depredated [43]. There has been no quantification of rodent predation on any Australian nesting birds, despite this information being essential for designing and applying evidence-based management [44]. Black rats are adept climbers, and anecdotal evidence suggests that they climb more than their native Australian counterparts [45]. This greater arboreal activity by black rats may allow them to be an added source of predation for arboreal-nesting birds and to limit nesting success rates.

Here, we test whether black rat predation is an added source of mortality in our study system using three approaches. We first use a spool and line method to quantify arboreal habitat use by bush rats and black rats, and then use manipulation experiments to quantify nest predation in sites where black rats were removed compared to untreated sites. We then compare the empirical responses derived from our manipulation experiments with benchmarks derived from Salo et al. [46] who found that exotic predators have on average twice the impact of native predators on prey populations. Our expectation is that the effect size of exotic rodents on artificial bird eggs will be similar to that of an exotic predator because black rats are arboreal rodent predators whereas their locally extinct native counterparts rarely climbed.

## Materials and Methods

This work was conducted under Scientific License no. SL100174 from the NSW Department of Environment and Heritage. Animal Care and Ethics approval was granted by the University of Sydney (L04/6-2011/3/5549). Experiments were carried out under the Australian Code for the Care and Use of Animals for Scientific Purposes. Great care was taken to minimise disturbance to animals. We used isoflurane to reduce handling stress of rats during trapping and spooling procedures, and ensured that spooled individuals carried less than 5% additional weight relative to their body mass.

### Study sites

The study was undertaken in Sydney Harbour National Park (SHNP; 33.8°S, 151.2°E), Sydney, Australia. Black rats are the most abundant small mammal within the National Park and surrounding areas; bush rats (and most other small mammals) are locally extinct [37]. Research was conducted in the Austral spring (October and November 2011) when native birds nest, and in conjunction with a small-scale bush rat reintroduction program. This reintroduction allowed us to evaluate the climbing behaviours of the two species in comparable habitat remnants. We used 16 sites in habitat remnants of the SHNP. Each site consisted of 36 grid points used for systematic deployment of nests and/or traps, spaced in a 6 x 6 array with consecutive grid points 20 m apart, and with all sites separated by > 1 km in different remnants to ensure independence (mean home range of local black rats = 1.05 +/- 0.08 ha). Local vegetation communities across the sites consisted of tall woodland (dominant species: *Angophora costata*; *Eucalyptus piperita*) that transitioned to Eastern Suburbs Banksia Scrub in the east (*Leptospermum laevigatum*, *Banksia aemula*, *Monotoca elliptica*). Vegetation condition varied across the sites, with some sites being more degraded than others (e.g. with infestations of exotic *Lantana camara*, *Ligustrum lucidum*).

### Rodent manipulations

We used live trapping to census or manipulate rodent numbers on the study sites for three months before beginning our nest predation experiment (November 2011). We had three rat density treatments: (i) control sites (hereon termed ‘unmanipulated’, n = 8 sites), where rat numbers remained unmanipulated and black rats were in high density; (ii) ‘removal’ sites (n = 4), where black rats were continually removed by trapping for 3 nights once a month; and (iii) ‘reintroduction’ sites (n = 4), where black rats were removed and bush rats were introduced from a source population located in similar habitat outside Sydney. Bird egg experiments were only conducted on two of these rat density treatments (control and removal).

Prior to the bush rat reintroduction, all removal and reintroduction sites were trapped for 10 days and black rats were removed and euthanased (initial black rat density across these sites was 17.0 ± 3.9 black rats/ha; error values reported in this paper are all standard error of the mean). Twenty-five bush rats were translocated to each reintroduction site in August 2011. Unmanipulated sites had on average 40.0 ± 7.15 black rats/ha at this time, whereas removal sites had, at most, one or two untrapped black rat individuals. The relevant rodent densities on treatment sites at the time of the bird egg experiment are reported below in the results section.

### Spool and line technique

We used spool and line tracking [47] to estimate the extent of bush rat and black rat activity on the ground and in trees (analogous to [48]). We collected and processed 11 black rats and 9 bush rats from reintroduction sites and 10 black rats from unmanipulated sites between dusk and 1.00 am over the course of a month (July 2012). Individuals were handled under a light anaesthetic (isoflourane) while they were weighed, sexed and fitted with spools. We used nylon spools (32 mm x 10 mm) with a maximum spooling distance of c. 160 m, covered in masking tape to ensure that the thread was not directly attached to the individual, and attached to avoid disrupting animals’ natural movements. Spool units weighed < 4 g (i.e. < 5% body weight) and were attached to a shaved area of the rump using non-toxic adhesive (cyanoacrylate superglue). Upon release, we tied the spool end to nearby vegetation, placed the rodent in its handling bag (except for the rump with the attached spool to prevent entanglement) under cover to reduce perceived and/or real predation risk, and left the animal to emerge from the handling bag in its own time to reduce flight effects.

The following morning we followed the spool lines. The spool thread catches on twigs, branches, leaves and other obstructions where it has unravelled, and thus is naturally split into sections. We measured the length of each of these spool sections to the nearest mm and classified sections of spool as ‘arboreal’ if they were > 1.5 m above ground (the minimum nest height of small nesting birds in Sydney); this is also approximately the same height used in previous studies on artificial nest predation [41, 49]. If a section of line crossed into the arboreal zone, it was recorded as two lengths: one at ground level and one in the arboreal zone. On the few occasions where the spool length passed out of sight, or was too high to measure, we estimated distance by projecting the spool length into an area that we could measure with a tape.

### Nest predation

Artificial nests are commonly used to quantify relative predation rates [50] because they overcome ethical issues associated with testing predation on real nests. While artificial nests have limitations [51–53], they can still be regarded as a valid indicator of the *relative* effect of an experimental treatment. In our case, we changed only black rat density between sites and examined nest predation rates. The approach of comparing relative rates of artificial nest predation between treatments has been used effectively in previous studies [42, 50].

We used artificial nests to quantify relative differences in predation between sites (unmanipulated vs control treatments) and to identify egg predators from tooth imprints left on plasticine eggs [40]. We used nests (65 mm diameter, 30 mm high) that were typical of those constructed by local small-medium sized birds by coating half-tennis balls with coconut husk fibre using a non-toxic glue, and securing two small wooden rods (diameter 6 mm, length 40 mm) parallel to the base of the nest to keep the nest stable on shrub or tree branches (see [40, 54]). We drilled two additional holes into the base of the nest to secure the eggs, and attached painted brown garden-ties to the nests. The ties were then used to secure nests to branches in the field. We modelled eggs from non-sulfurous plasticine (Rainbow modelling clay, Newbound P/L) to represent eggs of a common local bird species, the New Holland honeyeater (*Phylidonyris novaehollandiae*). Eggs were made using a silicon mould, and were secured in the tennis-ball nests using a garden-tie that passed through the centre of each egg; this made it hard for nest predators to completely remove eggs from the nests.

Black rats, like other mammalian predators, are excellent at distinguishing small differences in complex chemical odour cues [42, 55, 56] and use scent to hunt for eggs at night. We simulated prey scent using quail (*Coturnix japonica*) odour. The amount of odour used was consistent between sites, and hence any differences in egg survival would have resulted from differences in black rat density rather than changes in prey cue. Each nest was deployed in the field with an additional domestic quail egg and about 10 g of quail manure to provide semi-realistic olfactory cues for predators (see [42]). The quail manure was stored frozen and applied only once at the start of the experiment; treating manure this way does not alter its attractiveness to black rats (see [56]). All nests and eggs were handled using latex gloves to limit any confounding anthropogenic odours and to reduce olfactory recognition by potential nest predators.

This experiment was carried out in two blocks of two weeks on six sites at a time (two sites per treatment) in Austral spring, 2011. We deployed 36 nests on each site (36 points per site; 12 sites in total; four removal and eight unmanipulated sites) and left nests in place for 14 days; this is the average incubation period for New Holland honeyeaters [57], and the typical incubation period for other small local birds such as fantails, robins and honeyeaters [58]. As the average territory size for a New Holland honeyeater pair is 528.3 m^2^ [59], and birds have been observed to nest 25 m apart [60], our deployment of 1 nest per 20 m x 20 m (i.e. 400 m^2^) is within the upper limit of the natural expected density. We deployed nests in suitable habitat ≥ 1.5 m above ground, within the typical range of nest heights for New Holland honeyeaters [61], and the same height definition that we used to define arboreality for bush and black rats. We classified suitable nesting habitat as a tall shrub or tree with a well-covered nesting area, and a vertical branch or patch of branches where the nest could be stably secured. We secured nests to trees and inspected them after one, two, four, eight and 14 days or until the nest was attacked. A predation event was defined when the quail egg was either damaged or missing and/or the plasticine egg was disfigured. If we found that only the quail egg had been attacked, then we classified the predator as ‘unknown’. In all other cases, we inferred the identity of the nest predator by (i) examining bite marks on the plasticine eggs, (ii) making visual comparisons of bite marks with the conformation of teeth in reference skulls, (iii) comparing bite marks with images caught on infra-red cameras (ScoutGuard^®^ SG550V-5MP Compact Trail Security Camera) in pilot trials, and (iv) using previous studies as guides [40, 62]. The most common bite marks that we identified included those from birds, common brushtail possums (*Trichosurus vulpecula*), common ringtail possums (*Pseudocheirus peregrinus*) and rodents. We could not distinguish between bush rats and black rats using bite marks.

### Statistical analyses

We analysed all data using the statistical programs JMP^®^ version 9.0.0 [63] and R version 2.4.1 [64], and tested model fits for residual normality and homogeneity of variances using the Shapiro-Wilk-W test and Bartlett’s test, respectively. We transformed data to meet these assumptions, where appropriate, but failing that, we used alternative modelling approaches with more flexible distribution assumptions as noted below.

To analyse the spooling data we used generalised linear mixed models in R to test if black rats and bush rats exhibited different amounts of activity in trees, using ‘percentage of spool line in tree’, where tree was defined as spool found > 1.5 m above the ground, as a proxy for time spent climbing. Since our spooling data failed tests of normality and homogeneity of variance, we used a negative binomial distribution in the *glmmadmb* package, with ‘percentage of spool line in tree’ as the dependent variable, and ‘rodent species’ and ‘site’ as fixed and random independent variables, respectively. We tested if there was a difference in the arboreality of bush and black rats by comparing the full and reduced models (with and without ‘rodent species’ respectively) using the approximation that the log-likelihood ratio (LRT; defined as twice the difference between the log-likelihood value of the full minus the reduced model) equals the chi-squared distribution [65].

To analyse rodent trapping data, we used generalised linear models to compare the numbers of black rats known to be alive on removal and unmanipulated sites. ‘Site’ was again included as a random variable. This analysis was necessary to confirm that our treatment procedures did in fact change black rat densities on treatment sites as we intended.

We then compared the total percentage of non-depredated eggs between removal and unmanipulated sites after 14 days using a restricted maximum likelihood (REML) ANOVA with ‘treatment’ as a fixed factor and ‘site’ as a random factor. We repeated the same procedure for the total percentage of nests attacked by birds only, and then again for rats only after 14 days. The rat predation data were square root transformed to account for unequal variances. We calculated the effect size of the total surviving egg population after predator manipulation using the program METAWIN version 2.1 [66]. Finally, we compared our effect sizes to those for exotic and native predators calculated by Salo et al. [46].

## Results

### Black rat and bush rat use of arboreal areas

**Fig 1:**
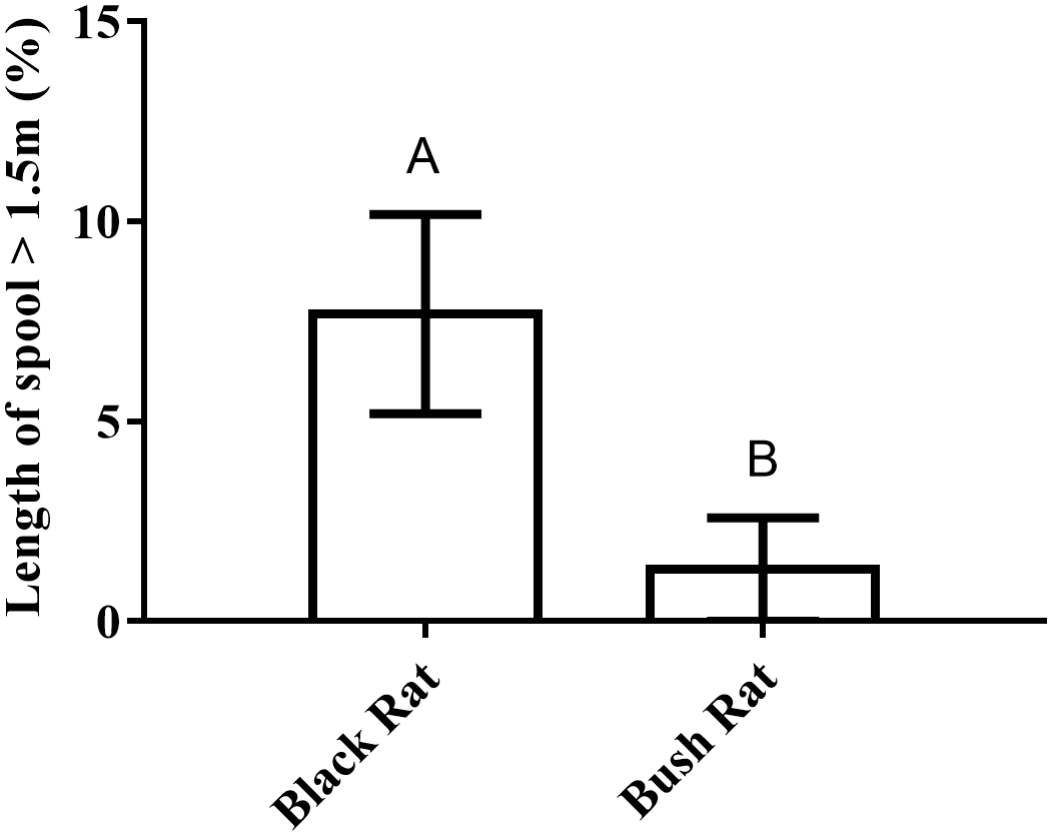
Arboreality of bush rats and black rats. A comparison of the arboreal activity of black and bush rats, shown as the average percentage length of spool left in trees (where arboreality is defined by spool left > 1.5 m above the ground) by both species. Error bars represent standard error, and different letters indicate that means differ with *p* < 0.05.

Black rats left relatively more spool line in trees compared to bush rats (*z*_(*n* = 21)_ = −5.24, *p* < 0.001, Fig 1). Including the fixed factor ‘rodent species’ significantly improved the model used to explain the amount of spool left at > 1.5 m above the ground ($\chi_{1}^{2}\;=\;36.7,\;p<0.001$); that is, species type was an important indicator of how much an individual climbed.

### Rodent manipulations

At the time of the nest predation experiment, the minimum numbers of black rats known to be alive were fewer on the removal sites (1.80 ± 0.81 individuals/ha) than on the unmanipulated sites (11.81 ± 2.35 individuals/ha) ($\chi_{1}^{2}\;=\;17.8,\;n\;=\;12,\;p<0.001$). Reintroduction sites had 12.88 ± 3.34 black rats/ha and 13.25 ± 1.92 bush rats/ha, and there was no difference between the number of black rats on unmanipulated and reintroduction sites.

### Nest predation

**Fig 2:**
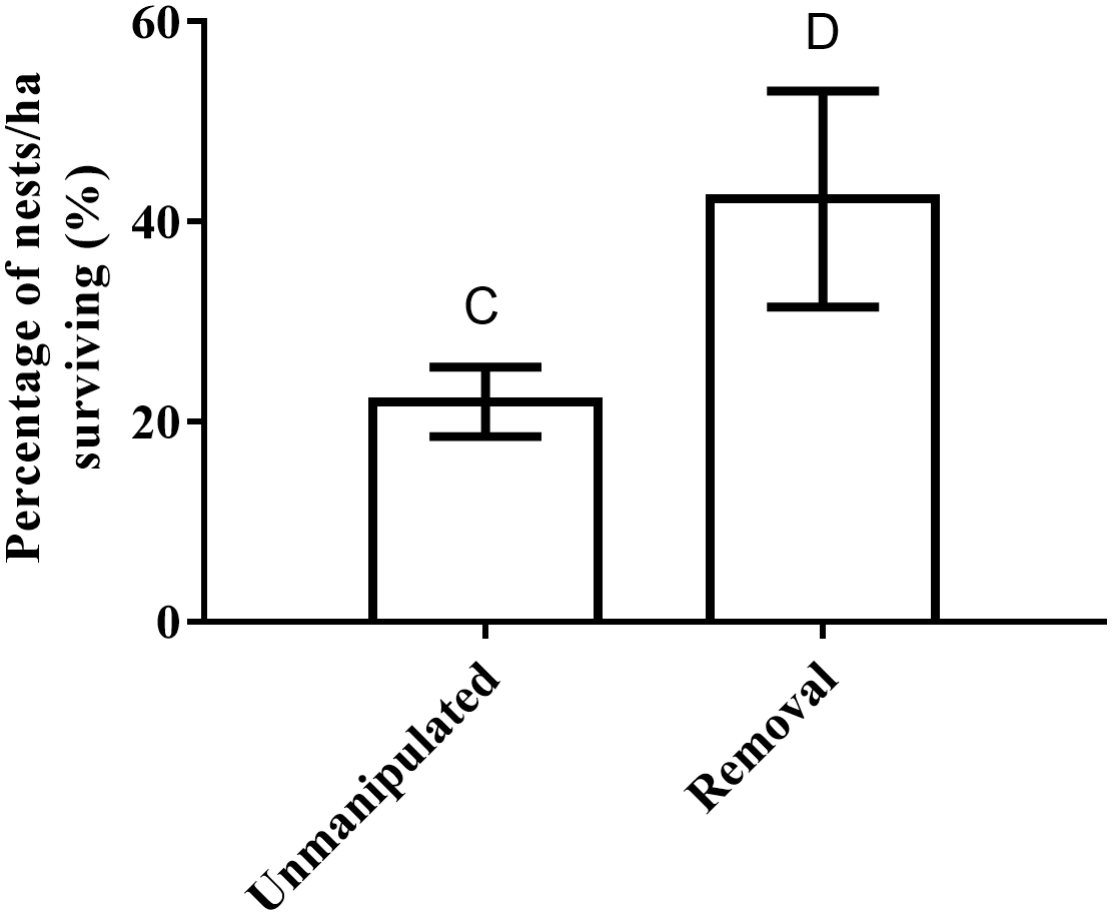
Nest survivorship. Average percentage of nests surviving with eggs intact per 1-ha site after 14 days. Error bars represent standard error and different letters represent treatments (unmanipulated or black rats removed) with means that are significantly different at *p* < 0.05.

**Fig 3:**
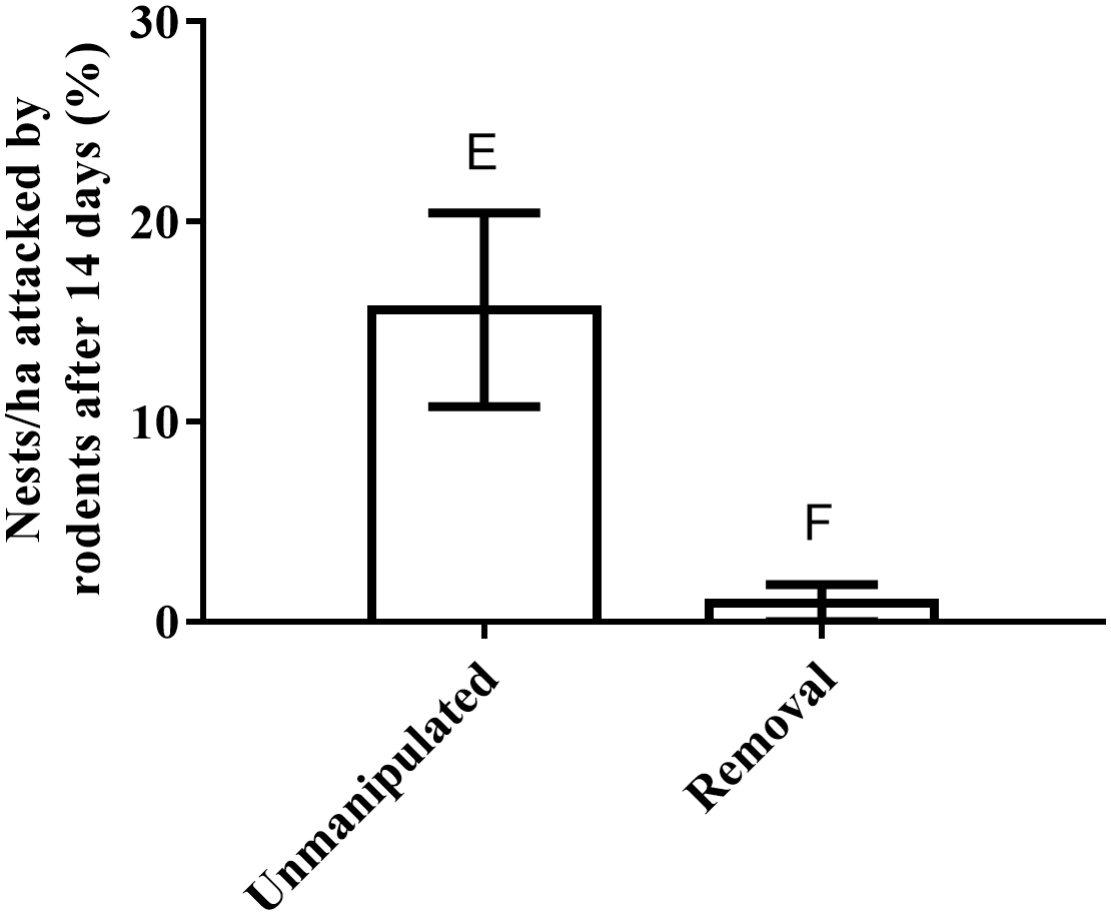
Nest predation by rodents. Average percentage of nests with eggs attacked per 1-ha site by rodents after 14 days. Error bars represent standard error and different letters represent treatments (unmanipulated or black rats removed) with means that are significantly different at *p* < 0.05.

After 14 days, the total number of eggs surviving on removal treatments was higher than on unmanipulated treatments (*F*_(1,10)_ = 5.29, *p* = 0.04, Fig 2). The total number of eggs attacked by rodents on removal sites was also lower than on unmanipulated sites (*F*_(1,10)_ = 7.22, *p* = 0.02, Fig 3), but there was no difference in the percentage of eggs attacked by birds on removal and unmanipulated sites (*F*_(1,10)_ = 2.12, *p* = 0.17). Around half of all attacks on eggs were from birds: 56.7 ± 5.0% of eggs were attacked by birds on unmanipulated sites (n = 162 nests), compared with 48.6 ± 7.8% on removal sites (n = 69 nests). Less than 20% of nest attacks were made by rats: 14.0 ± 3.8% of eggs were attacked by rats on unmanipulated sites (n = 35 nests), compared with a single nest on removal sites. Less than 10% of nest attacks were made by brushtail possums: 4.8 ± 2.6 on unmanipulated sites (n = 12 nests), compared with 4.7 ± 3.2 on removal sites (n = 5 nests). The remainder of attacks included ringtail possum, ants and unidentified markings.

### Effect size

We calculated an effect size of 1.3 using the total number of eggs surviving after 2 weeks on unmanipulated and removal sites. When compared to the mean effect size estimates of Salo et al. [46], calculated from analogous experiments on prey responses to removals of native and introduced predators, our effect size estimate of 1.3 falls within the expected range (1.224–3.046) for an ‘exotic’ predator (see Fig 1 of [46]).

## Discussion

This study is the first to quantify the predatory impacts of black rats on arboreal nesting birds in Australia in a system where black rats have replaced native rodent counterparts. Consistent with our predictions, black rats contributed significantly to nest attacks, and therefore appear likely to contribute an additive source of nest mortality for small birds in urban habitat remnants.

### Black rats are more arboreal than native bush rats

Black rats left approximately five times more spool line in trees than bush rats, thus supporting previous conjecture that black rats use the arboreal zone more than bush rats. Black rats are well known for their arboreal activity [67], but our spooling data quantify for the first time the minimal arboreal activity of bush rats. Differences in climbing ability between two potentially scansorial congeners are often attributed to weight and dominance, where the smaller subdominant species tends to climb more than the larger dominant species [68, 69]. However, bush rats and black rats are similar in mass, occupy similar habitat [70–72] and are thought to be competitively symmetrical [34]. Instead, key differences in morphology, such as the longer tail of the black rat compared to the bush rat, more likely explain the differences in arboreality by conferring black rats with greater balance and agility above ground [45] than their congeners.

Being substantially more arboreal than their native counterparts, in a system where black rats have replaced bush rats, exotic black rats could impose an additive source of predation on arboreal nesting birds, as well as on other taxa such as invertebrates [73–76] and bats [77–79]. Arboreal predation may in turn induce behavioural changes in some species, and/or threaten the survival of others (e.g. [7]). Black rats may also compete with native species for arboreal resources (e.g. hollow-dwelling species [80]). Hooker and Innes [81] found that black rats in New Zealand forest habitats nest exclusively in trees and sometimes in hollows, and similar observations have been made in Australia [82]. In urban habitat remnants, where black rats are common, hollows are a scarce resource due to severe clearing of large trees [80], and black rats may add to the competitive pressures on hollow-dwelling species for nesting/roosting space [21].

### Black rats are nest predators

We found fewer rodent bite marks on eggs in black rat removal sites compared with those from unmanipulated sites, thus supporting our hypothesis that black rats represent an additive source of nest mortality to cup nesting birds by black rats in a system where black rats have replaced native *Rattus* spp. Nest predation is frequently cited as the main and most common cause of nest failure [6–9], and our study is consistent with previous studies in the area in finding that predation by birds is substantial [40, 83]. In urban habitat remnants, where other processes such as habitat loss and modification and redistribution of resources also contribute to nest failure, rodent nest predation has significantly negative effects on arboreal small bird species.

Nest predation is high in areas with high numbers of nest predators [84, 85], although this relationship can vary spatially and temporally and with local and regional environmental characteristics [39, 86, 87]. For example, our results differ from those of Matthews et al. [40] who found that rodents were not significant predators of bird eggs in another part of Sydney (< 10% of eggs at their sites had rodent bite marks). The habitat remnants used by Matthews et al. [40] were much larger than our sites (a third of the sites used by Matthews et al. [40] were > 100 ha) and the densities of black rats was not reported but may have been lower than our sites if native rats were still common.

Nest-raiding birds were the main predators of our nests, which is similar to findings from other artificial nest studies in Australia [40, 41], including studies on ground-nesting birds [88]. Large, aggressive birds often depredate smaller birds, nestlings and eggs [41, 61, 84, 89, 90]. These predatory species often succeed well in urban areas [91–94] due to reduced predation (larger, higher-order predators are locally very scarce or extinct) and their ability to exploit human resources [95]. In our study system, candidate predators include laughing kookaburras (*Dacelo novaeguineae*), pied currawong (*Strepera graculina*), and other corvids (e.g. *Corvus coronoides*). For example, McFarland [61] observed kookaburras and pied currawongs depredating New Holland honeyeater fledglings under natural conditions. These large birds are increasing in numbers in urban areas (e.g. [96]), and this is likely to limit populations of smaller resident birds [94]. In combination, predation pressure from exotic commensal rodents plus native large birds likely has highly negative impacts on small nesting birds in urban remnants.

Rates of nest predation by birds was not affected by our experimental manipulation of black rat density. Thus the decline in all nest predation events on removal sites was driven by a reduction in direct rodent predation rather than via an indirect suppression of large bird predators.

### Black rats act as exotic nest predators

Black rats were a significant source of bird egg predation on our unmanipulated sites, where there were high densities of black rats. Our calculated effect size (1.3) fell within the range of that expected for an exotic predator [46], supporting our suggestion that black rats are not only a significant source of predation for small tree-nesting birds but have an exaggerated effect over that of native predators. Salo et al. [46] also identified the effect size of exotic predators as being more than three times that of native predators in Australia, although this result was based on only three studies [97–99], all of them involving foxes. Therefore, using the most conservative estimate that pools all exotic predator manipulation studies together, we still find that our effect size for the black rat equates with that for an exotic predator. The most parsimonious explanation for this exaggerated effect is that exotic black rats represent a novel tree-climbing rodent predator in this system, with native birds more likely to have co-evolved strategies to defend against other native predators and rodent predators that spend little time above ground.

We found that introduced black rats imposed significant damage on the artificial eggs and nests that we used. We suggest from this that black rats represent an additive source of predation for nesting urban birds, although longer-term experiments are needed to determine if predation is additive or simply compensates for other sources of mortality that might occur later, as might be expected, for example, under the doomed surplus hypothesis [97]. We predict that black rat impacts on eggs and nests are likely to flow on and have population-level impacts on native birds, and that depredation from black rats in part explains why small native birds have declined in Sydney and elsewhere [100, 101]. In our study system, the loss of small birds has been attributed to fragmentation effects [40, 102] and human subsidies which indirectly increase the abundance of other aggressive and predatory native species [103, 104], in particular native miners and introduced Indian Mynas [105]. In urban areas in general, commensal species such as cats and dogs have also been implicated in the loss of native birdlife. However, commensal rodents have seldom been considered as an important driver of bird populations and assemblages in urban and peri-urban bushland remnants [21]. Our results suggest that commensal rodents have the potential to significantly contribute to other pressures of urban living for native birds, and that the management of commensal rodents must be considered in recovery plans for urban birdlife.

### Management implications

Our results have strong implications for the conservation of wild nesting birds in urban habitat remnants, or in fact any other prey of black rats that emit strong odour cues. More work around effective chemical camouflage of vulnerable prey species may prove an effective approach for confusing olfactory predators and reducing predation rates on native species [42].
